# No evidence that plasmablasts transdifferentiate into developing neutrophils in severe COVID‐19 disease

**DOI:** 10.1002/cti2.1308

**Published:** 2021-06-30

**Authors:** José Alquicira‐Hernandez, Joseph E Powell, Tri Giang Phan

**Affiliations:** ^1^ Garvan Institute of Medical Research Darlinghurst NSW Australia; ^2^ Institute for Molecular Bioscience University of Queensland St Lucia QLD Australia; ^3^ UNSW Cellular Genomics Futures Institute University of New South Wales Sydney NSW Australia; ^4^ St Vincent’s Clinical School Faculty of Medicine UNSW Sydney Darlinghurst NSW Australia

**Keywords:** B cells, COVID‐19, emergency granulopoiesis, low‐density neutrophils, neutrophils, plasma cells, SARS‐CoV‐2, single‐cell RNA sequencing

## Abstract

**Objectives:**

A recent single‐cell RNA sequencing study by Wilk *et al*. suggested that plasmablasts can transdifferentiate into ‘developing neutrophils’ in patients with severe COVID‐19 disease. We explore the evidence for this.

**Methods:**

We downloaded the original data and code used by the authors in their study to replicate their findings and explore the possibility that regressing out variables may have led the authors to overfit their data.

**Results:**

The lineage relationship between plasmablasts and developing neutrophils breaks down when key features are not regressed out, and the data are not overfitted during the analysis.

**Conclusion:**

Plasmablasts do not transdifferentiate into developing neutrophils. The single‐cell RNA sequencing is a powerful technique for biological discovery and hypothesis generation. However, caution should be exercised in the bioinformatic analysis and interpretation of the data and findings cross‐validated by orthogonal techniques.

## Graphical abstract

Single cell RNA sequencing can generate unexpected hypotheses. However, these should be rigorously tested and cross‐validated by orthogonal techniques. In this case, there is no evidence that plasmablasts differentiate into neutrophils in severe COVID‐19 disease.

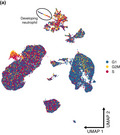

A recent study by Wilk *et al*. of the transcriptome of peripheral blood mononuclear cells (PBMCs) in seven patients hospitalised with COVID‐19 described a population of ‘developing neutrophils’ that were ‘phenotypically related by dimensionality reduction’ to plasmablasts and that these two cell populations represent a ‘linear continuum of cellular phenotype’.^1^ The authors suggest that, in the setting of acute respiratory distress syndrome (ARDS) secondary to severe COVID‐19, a ‘differentiation bridge from plasmablasts to developing neutrophils’ connected these distantly related cell types. This conclusion is controversial as it appears to violate several basic principles in cell biology relating to cell lineage identity and fidelity. Correctly classifying cells and their developmental history is an important issue in cell biology, and we suggest that this conclusion is not supported by the data as we show here that (1) the ‘differentiation bridge’ between plasmablasts and ‘developing neutrophils’ is not replicable in other patients with mild and severe COVID‐19; (2) accounting for cell cycle stage disrupts the plasmablast–neutrophil relationship; (3) regressing out covariates such as unique molecular identifiers (UMIs) can lead to overfitting of the data; and (4) UMAP embeddings may reflect the expression of similar genes by different cell clusters but not necessarily direct cell lineage relationships between them.

Infection‐induced plasmablasts are proliferating plasma cells derived from B cells of the lymphoid cell lineage that secrete antibodies against invading pathogens.^2^ In contrast, neutrophils are cells derived from the myeloid cell lineage that trap, phagocytose and kill pathogenic organisms by generating reactive oxygen species, secreting proteases and degradative enzymes and casting neutrophil extracellular traps (NETs).^3^ Accordingly, the lineage conversion of plasmablasts into developing neutrophils requires a massive reorganisation of the cell at the level of the transcriptome, epigenome and proteome. While the forced transdifferentiation of B‐cell lines, B‐cell progenitors and naïve B cells into macrophages has been described, notably via the ectopic expression of CCAAT/enhancer‐binding protein (C/EBP) family of transcription factors,^4^, ^5^ the spontaneous transdifferentiation of terminally differentiated plasma cells is an entirely different hypothesis that would require compelling evidence.

The recent introduction of high‐throughput single‐cell RNA sequencing (scRNA‐seq) has created new opportunities for developmental biologists to map the developmental history of differentiated cell types.^6^ These technologies assay the expression of genes in a large number of cells – their cell transcriptional state – and a standard computational approach is used to flatten these high‐dimensional data into a two‐dimensional (2D) Euclidean space as state manifolds. In these projections, the proximity of one cell type to another may denote similarities in the cell transcriptional state. A major challenge in the analysis of scRNA‐seq data is to remove noise from technical variation while preserving biological heterogeneity.^7^ To understand how this trade‐off is balanced, we first used their code to reproduce Figure 1c from their paper (Figure 1a). Likewise, we analysed a reference data set of 99,049 PBMCs comprising 8 mild and 10 severe COVID‐19 patients, and 22 control samples produced by Schulte‐Schrepping *et al*.^8^ to study alterations in the myeloid cell compartment in patients with severe COVID‐19 (Figure 1b). We integrated both Wilk’s and Schulte‐Schrepping’s data sets and examined their joint cell‐type relationships in a common UMAP space. Notably, we observed that the ‘developing neutrophils’ are no longer related located adjacent to plasmablasts as observed by Wilk *et al*. but instead were positioned closer to neutrophils (Figure 1c). Indeed, ‘developing neutrophils’ resemble the population of immature neutrophils reported by Schulte‐Schrepping *et al*. in their study, which create a phenotypic continuum with mature neutrophils (Figure 1d). Moreover, mature neutrophil populations were also concordant between both studies. These results suggest that the presence of a ‘differentiation bridge’ between plasmablasts and neutrophils is highly unlikely and the phenotypic relationship between developing neutrophils observed by Wilk *et al*. could be instead driven by: (1) normalisation and modelling of data and (2) the presence of shared cell states between cell types confounding phenotype similarities.

**Figure 1:**
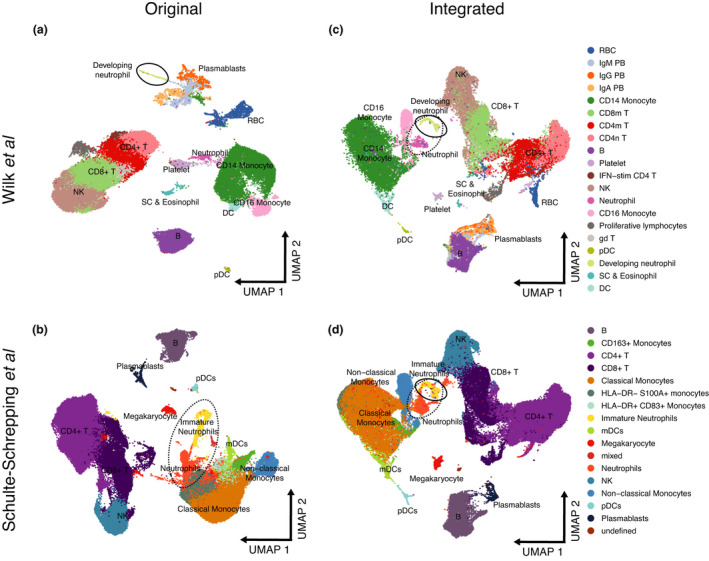
Re‐analysis of scRNA‐seq data from Wilk *et al*. shows no supporting evidence for the hypothesis that ‘developing neutrophils’ transdifferentiate from plasmablasts. **(a)** Reconstruction of the UMAP embedding from Figure 1c of Wilk *et al*. **(b)** Reconstruction of the UMAP embedding from Figure 2a of Schulte‐Schrepping *et al*. **(c)** Integrated Wilk *et al*.*’s* and Schulte‐Schrepping *et al*.*’s* data sets. ‘Developing neutrophil’ embeddings are closer to neutrophils and the myeloid cell compartment, while plasmablasts remain associated with B cells. **(d)** Integrated Wilk *et al*.*’s* and Schulte‐Schrepping *et al*.*’s* data sets. ‘Developing neutrophils’ from the Wilk *et al*. study **(c)** colocalise with immature neutrophils in this cohort.

To test these hypotheses, we first noted that, in addition to regressing out mitochondrial genes, ribosomal RNA and ribosomal genes, the authors also regressed out the number of UMIs (nCount_RNA) and the number of expressed genes (nFeature_RNA) from the gene expression data in their analysis. This step is unnecessary in Seurat when using the SCTransform normalisation method as the number of UMIs is explicitly modelled using a regularised negative binomial regression.^7^ Regressing out the number of UMIs using a standard generalised linear model (GLM) is discouraged by the developers because of overfitting.^7^ Moreover, the number of expressed genes in each cell is correlated with its number of UMIs (Pearson’s correlation = 0.9249). We therefore reanalysed the data in three ways: following the original analysis; regressing out only mitochondrial genes, potentially associated with cell quality (Figure 2b); and regressing out mitochondrial and ribosomal genes, the latter not commonly accounted for in single‐cell analyses (Figure 2c). This exercise shows that the reported relationship between developing neutrophils and plasmablasts breaks down when technical noise is not removed at the expense of biological variability and that the data are not overfitted.

**Figure 2:**
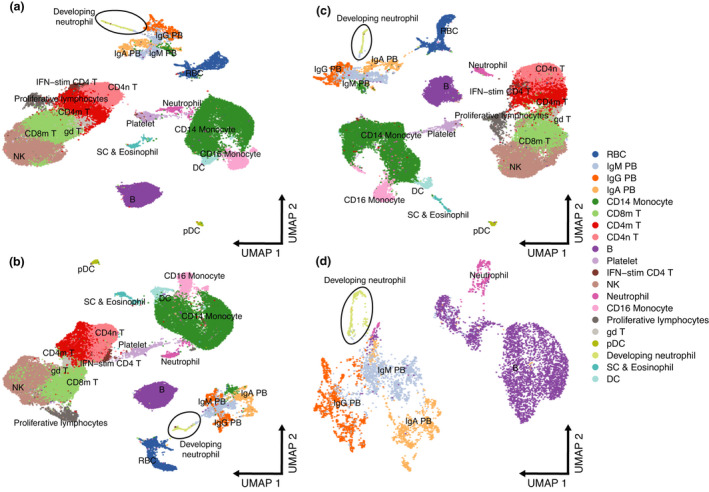
Effect of regressing out biological covariates in the Wilk *et al*. data set. **(a)** Original UMAP embedding from Figure 1c of Wilk *et al*. **(b)** UMAP embedding showing effect of regressing out mitochondrial genes. **(c)** UMAP embedding showing effect of regressing out mitochondrial genes and ribosomal genes. **(d)** UMAP embedding of neutrophils, developing neutrophils, B cells and plasma cells regressing out mitochondrial genes only.

Accordingly, we believe that orthogonal approaches, such as single‐cell DNA sequencing to detect rearranged immunoglobulin heavy‐ and light‐chain variable genes in the developing neutrophils, are needed to provide evidence of their B‐cell origin and support the hypothesis that terminally differentiated plasma cells transdifferentiate into developing neutrophils. Furthermore, unless the data visualisation parameters are carefully chosen, the 2D UMAP embeddings may give the misleading impression that cell clusters are closer or further than they actually are. For example, in Figure 1c of Wilk *et al*., developing neutrophils appear to occupy a similar manifold space as plasmablasts. However, from this viewpoint the plasmablasts appear to be distantly related to the B cells from which they are derived. In addition, these types of analyses make the *a priori* assumption that all the cell types being studied are developmentally related and linked by a cell lineage tree. This may hold true when analysing *in vitro*‐differentiated cells that share a common ancestor cell of origin that is present in the cell culture, but may not apply to PBMCs that contain a heterogeneous collection of cell types that have arisen from different committed progenitors.

Since plasmablasts are derived from B cells, we decided to subset neutrophils, developing neutrophils, B cells and plasmablasts to further explore any similarities between these cell clusters. This analysis revealed that counter‐intuitively, neutrophils were not related to developing neutrophils and that B cells were not related to plasmablasts (Figure 2d). This exercise suggests instead that the developing neutrophils and plasmablasts, in responding to severe COVID‐19 disease, are activated cell types that may share the expression of a number of genes and gene modules that have led to their misclassification as related cell types. These gene modules may result from cellular proliferation and hyperactivation of the immune system by SARS‐CoV‐2. Accordingly, we evaluated the effect of cell cycle on the plasmablast‐developing neutrophil relationship and observed a disruption of their relationship when cell cycle is taken into consideration (Figure 3a and b). To identify conserved gene modules between the two cell types, we performed logistic regression using the gene expression as the explanatory variable and the cell type as the response for each gene expressed and obtained the AUROC for each gene and selected the least informative 25% genes. Gene ontology enrichment analysis for these conserved genes confirmed that the majority were indeed biological processes related to the cell cycle (Figure 3c).

**Figure 3:**
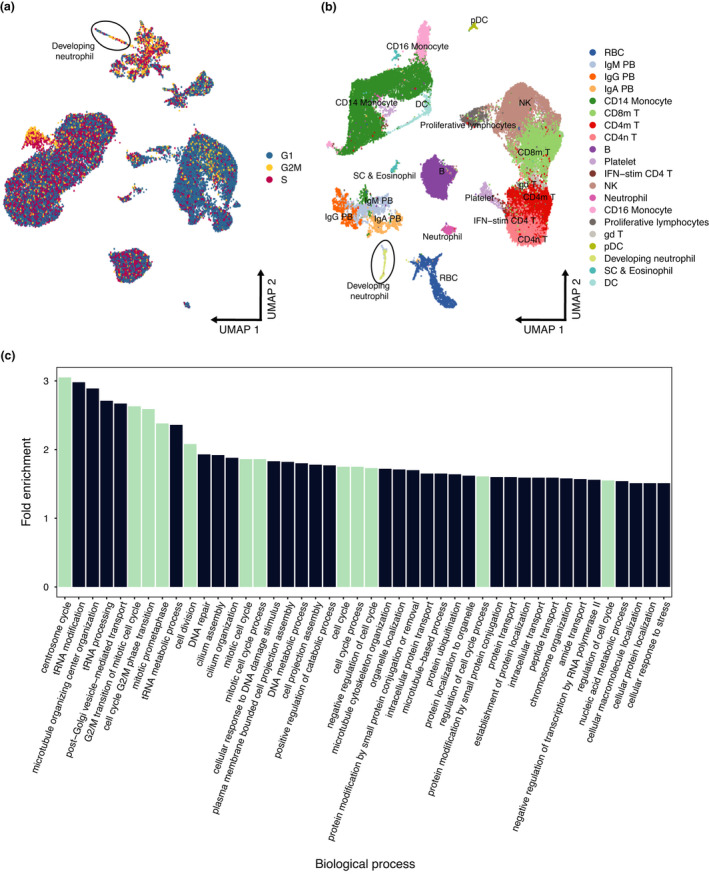
Effect of regressing out cell cycle from the Wilk *et al*. data set. **(a)** Original UMAP embeddings showing the inferred cell cycle phase. Plasmablasts and ‘developing neutrophils’ are found in multiple cell cycle stages. **(b)** UMAP embedding showing the effect of regressing out cell cycle signals from gene expression data. ‘Developing neutrophils’ separate from the plasmablast subpopulations. **(c)** Gene ontology enrichment analysis of conserved genes between plasmablasts and ‘developing neutrophils’. Biological processes related to the cell cycle are shown in turquoise.

In their analysis, Wilk *et al*. point out that the developing neutrophils were unlikely to be doublets and also unlikely to be granulocytes that had phagocytosed B cells as there were no clinical features of haemophagocytic lymphohistiocytosis (HLH), a well‐characterised state of immune hyperactivation. However, HLH is often difficult to diagnose clinically and severe COVID‐19 disease has been linked in some cases with secondary HLH.^8^, ^9^

Nevertheless, the paper by Wilk *et al*. is a timely and valuable contribution to the understanding of how severe COVID‐19 disease reshapes the composition and activation state of cell clusters in PBMCs. Neutrophils have a high density and are not normally present in Ficoll preparations of blood used to isolate PBMCs. The developing neutrophils may therefore be similar to the pro‐inflammatory low‐density neutrophils that have been described in a number of autoimmune diseases^10^ and in severe COVID‐19.^8^, ^11^, ^12^ These low‐density neutrophils consist of hyposegmented immature neutrophils (band forms on the blood film) that are also produced during emergency granulopoiesis.^13^, ^14^ Thus, it is more plausible that the developing neutrophils are produced in the bone marrow from myeloid precursors in response to overwhelming infection with SARS‐CoV‐2.

In summary, scRNA‐seq is becoming an increasingly popular tool for dissecting cellular heterogeneity in complex biological systems. However, it does have its limitations and, in the example shown here, may mislead and generate interesting hypotheses that risk being erroneously accepted as conclusions without further cross‐validation. Our analysis of the data from Wilk *et al*. indicates that there is not enough evidence to suggest that developing neutrophils transdifferentiate from plasmablasts.

## Methods

Before data integration, we split the Schulte‐Schrepping *et al*. by data origin to obtain individual batches and applied SCTransform normalisation to each batch and the Wilk *et al*. data set. Next, we determined 3000 highly variable genes (HVGs) for integration across all data sets. We identified mutual nearest neighbour cells between data sets to define integration anchors and integrated the data by computing anchor integration matrices. Finally, we performed a principal component analysis (PCA) using the implicitly restarted Lanczos method and computed the first fifty principal components and embedded the principal components into a two‐dimensional space using the Uniform Manifold Approximation and Projection (UMAP).

To test the effect of covariates on the low‐dimensional data representations, we applied SCTransform normalisation and included different covariates in the second non‐regularised linear regression as follows: (i) using the percentage of mitochondrial expression and (ii) using the percentage of ribosomal and mitochondrial expression. Next, we performed PCA and UMAP dimensionality reduction as described above. Additionally, we subsetted all B cells, plasmablasts (IgM, IgA and IgG), neutrophils and developing neutrophils. Next, we applied SCTransform using the original covariates considered by Wilk *et al*.: percentage of ribosomal (percent.rps, percent.rpl and percent.rrna) and mitochondrial (percent.mt) expression and the number of counts (nCount_RNA) and genes (nFeature_RNA) per cell. Finally, we performed PCA and UMAP following the same strategy described in the previous section. All analyses and graphs were generated using the Seurat v4.^15^

We calculated cell cycle scores with the CellCycleScoring function using a set of 43 genes associated with the S phase and 54 with the G2 M phase. Similarly, for the assessment of covariate effects, we regressed out the scores for both phases from the gene expression data within the SCTransform function. Gene ontology enrichment analysis was performed using the PANTHER over‐representation test with Fisher’s exact test and the Bonferroni correction for multiple testing (http://pantherdb.org/).

## Conflict of interests

The authors declare no competing interests.

## Author Contributions

**Jose Alquicira‐Hernandez:** Conceptualization; Data curation; Formal analysis; Methodology; Visualization; Writing‐original draft; Writing‐review & editing. **Joseph Powell:** Conceptualization; Formal analysis; Supervision; Validation; Writing‐original draft; Writing‐review & editing. **Tri** **Phan:** Conceptualization; Formal analysis; Supervision; Writing‐original draft; Writing‐review & editing.
